# Aqueous extracts of tree peony petals: renin and angiotensin I-converting enzyme inhibitory activities in different colours and flowering stages

**DOI:** 10.1039/d2ra00516f

**Published:** 2022-03-10

**Authors:** Yifang Gao, Xixi Li, Xueting Liu, Wenqing Yang, Mengru Li, Jiaying Li, Fengjuan Li

**Affiliations:** Key Laboratory of Food Nutrition and Safety, Tianjin University of Science and Technology, Ministry of Education No. 29 13th Avenue, Teda Tianjin 300457 PR China lifj@tust.edu.cn +86-22-60601457 +86-22-60912453

## Abstract

Tree peony (*Paeonia suffruticosa* Andr.) is an ornamental and medicinal plant from China. Previous studies have detected novel blood pressure-regulating substances in this species, which potentiate its value of utilization. To explore these substances, the aqueous extracts of 7 different colours of tree peony petals were assessed for inhibitory activity on renin and angiotensin-converting enzyme (ACE). The results showed that the activity of dark-coloured samples was significantly stronger than that of light-coloured ones. Furthermore, the inhibitory activity of the red tree peony petals ‘Hong TaiYang’ on renin and ACE indicated a downward trend from bud compaction to the full opening stage. The antioxidant activities of the aqueous extracts, on one side, and the correlations between phenolics and flavonoids functionalities and total contents, on the other, were also evaluated. In this regard, the extracts of different samples had ABTS free radical scavenging capacities of 17.28–210.41 mg TE per g DW, DPPH radical scavenging capacities of 35.45–150.78 mg TE per g DW, iron ion reduction capacities of 16.66–150.77 mg TE per g DW, and total phenolic content of 23.94–150.78 mg GAE per g DW. Correlation analysis revealed that the renin and ACE inhibitory activities, the DPPH and ABTS free radical scavenging capacities, and the iron reduction ability of different sample extracts were positively correlated with total phenolic contents (*p* < 0.01). Finally, the aqueous phenolic compounds in the sample extracts tended to show strong renin and ACE inhibitory activities and therefore exhibit a potential auxiliary blood pressure control prospect.

## Abstract 2

Tree peony petals with different colours and flowering stages were assessed for renin/ACE inhibition. Dark-coloured samples showed higher inhibition than light-coloured ones, and a downward inhibitory trend was found from bud to full opening stage.

## Graphical abstract

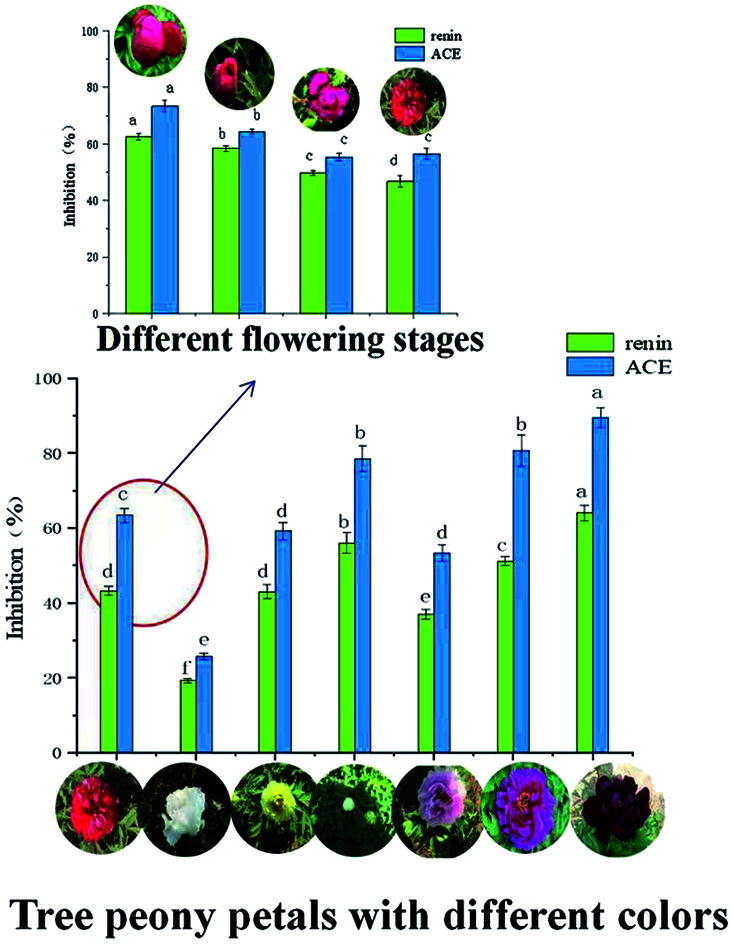

## 1 Introduction

Hypertension is the most common chronic disease and the main risk factor for cardiovascular and cerebrovascular diseases.^1^ The renin-angiotensin system (RAS) plays a crucial role in modulating blood pressure in the human body. Renin (EC 3.4.23.15), a rate-limiting enzyme, can hydrolyze the N-terminus of angiotensinogen to yield angiotensin I. Then, angiotensin I is further hydrolyzed to angiotensin II. Because of the catalysis of the angiotensin-converting enzyme (ACE; EC 3.4.15.1), these processes occur under strong vasoconstriction conditions that eventually increase blood pressure.^2^ Because of this, inhibiting the activities of renin and ACE is considered an effective way to prevent and treat hypertension. At present, synthetic inhibitors, such as aliskiren for renin and captopril and enalapril for ACE, have become popular drugs to treat the hypertensive. However, there are evident side effects associated with the usage of these inhibitors, like skin rashes, dry cough, as well as taste disturbance, which are thought to be inevitable.^3^ In this context, renin and ACE inhibitors derived from foodstuff have attracted the attention of researchers because they are easily absorbed and bear fewer side effects.^4^

Recently, many renin and ACE inhibitory peptides obtained from plant or foodstuff sources have been investigated, including bean hydrolysates,^5^ amaranth proteins,^6^ beef hydrolysates,^7^ flaxseed protein,^8,9^ and bovine fibrinogen.^10^ Although these food-derived peptides have provided new ideas for the prevention and treatment of hypertension, the efficacy of these peptides in the human body needs to be further verified. For example, some of them have been shown to easily decompose into inactive metabolites *in vivo*, which results in low bioavailability.^11^ Therefore, the research and development of safe and effective renin and ACE inhibitors is a present necessity.

Some clinical experiments have indicated that increasing daily intake of phenols has a beneficial effect in controlling hypertension.^12,13^ This could be due to the polyphenols reducing the oxidative damage that blood vessels receive. It is worth noting that some phenolic compounds derived from plants have been reported to inhibit renin or ACE. For instance, saponin from soybean,^14^ polyphenolic extracts of the green leafy vegetables *Vernonia amygdalina* and *Gongronema latifolium*,^15^ and tea polyphenols^16^ inhibit renin; on the other hand, polyphenols from tomato,^17^ flavonoids from the buds of rosa damascene,^18^ and polyphenols from Indian gooseberry^19^ inhibit ACE. In summary, these reports have shown that phenolics may regulate RAS by inhibiting renin and/or ACE and have a beneficial effect on the treatment of hypertension.

Tree peony (*Paeonia suffruticosa* Andr.), a woody deciduous shrub (Paeoniaceae family) native to China, is considered a traditional ornamental and medicinal plant.^20^ Now, tree peonies are cultivated all over the world, and more than 20 ha have been grown in China. Tree peony petals are beautiful and highly enriched in soluble sugars, flavonoids, anthocyanins, and gallic acid,^21^ which have become food materials and natural antioxidant resources to make yogurt, tea, cake, red wine, and essential oil.^22–24^ Recently, an increasing number of researchers have paid attention to the chemical components and biological activities of tree peony petals. Some papers have used advanced technologies to separate and identify phenols and flavonoids from tree peony petals and proved their antioxidant, anti-inflammatory, and antibacterial activities.^25,26^ In a previous study, we found that aqueous extracts of red and white tree peony petals had inhibitory activity on renin and ACE, but their inhibitory activity was significantly different.^27^ There is a diversity of colours among tree peony flowers with nine categories: red, white, yellow, green, pink, purple, black, blue, and dual colours. Different phytochemicals are attributed to each flower colour, and dark petals contain more anthocyanins and flavonoids.^21^ During flower growth, phenolic compounds such as the secondary metabolites, including anthocyanins and carotenoids, accumulate at different stage.^28^ Therefore, the content of bioactive substances varies with the flowering stage. In this study, we investigated inhibitory activity in aqueous extracts of tree peony petals. In particular, we considered 7 different flower colours and assessed renin and ACE activities. Additionally, renin and ACE inhibitory activity in aqueous extracts of red tree peony ‘Hong TaiYang’ at 4 different flowering stages was further valued. On the other hand, since oxidative damage in the blood vessels is closely related to the occurrence and development of hypertension,^12^ the antioxidant activity of the samples was also investigated. Other assessments included detection of total phenols and flavonoids and calculating correlations between the contents of total phenolics and their functionalities. This work could provide not only new ideas for exploring novel blood pressure regulatory factors but also a richer theoretical basis for further improving the functionalities of tree peony products with high-value utilization.

## 2 Material and methods

### 2.1 Materials

The human recombinant renin inhibitor screening assay kit, including the human recombinant renin and substrate (Arg-Glu(EDANS)-Ile-His-Pro-Phe-His-Leu-Val-Ile-His-Thr-Lys(Dabcyl)-Arg), was purchased from Cayman Chemical Co. (Ann Arbor, MI, USA). Angiotensin I-converting enzyme (ACE, from rabbit lung), *N*-hippuryl-His-Leu tetrahydrate (HHL), *o*-phthaldialdehyde (OPA), Folin–Ciocalteu phenol reagent, 1,1′-diphenyl-2-picrylhydrazyl (DPPH), 2,2′-azino-bis(3-ethylbenzothiazoline-6-sulfonic acid) diammonium salt (ABTS), 6-hydroxy-2,5,7,8-tetramethylchroman-2-carboxylic acid (Trolox), 2,4,6-tri(2-pyridyl)-*s*-triazine (TPTZ), gallic acid and catechin were sourced from Sigma-Aldrich (St. Louis, MO, USA).

**Fig. 1:**
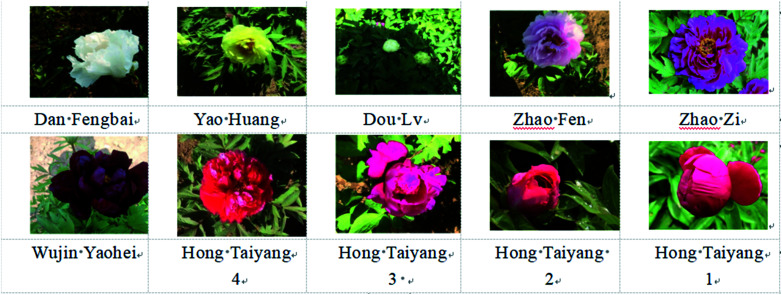
Names of the evaluated tree peonies. Red tree peony flowers ‘Hong TaiYang’ were classified according to flowering developmental stages.

The tree peony petals used in the experiments were collected from the Fuxi tree peony planting base of He Ze, Shandong, China. The petals of different colours including the red ‘Hong TaiYang’ were classified according to different flowering stages (Fig. 1). The tree peonies were transported on ice within 12 h, taken to a vacuum freeze dryer, and stored at −4 °C.

### 2.2 Preparation of sample extracts

All freeze-dried samples of petals were ground to a fine powder with a pulverizer, and each sample powder (0.5 g) was dispersed in 20 mL of distilled water. After mixing completely, the solution was centrifuged for 10 min at 5000 rpm. The supernatant was collected and filtered through 0.45 μM membrane filtration. The filtrate was recovered for the subsequent experiment.

### 2.3 Renin inhibition assay

The determination of renin inhibition was based on the method by Li *et al.*^16^ The human recombinant renin inhibitor screening kit was employed as follows: (1) blank: 20 μL of substrate, 160 μL of buffer, 10 μL of distilled water; (2) sample: 20 μL of substrate, 160 μL of buffer, 10 μL of sample solution. Then 10 μL of renin enzyme solution were added to the control and sample wells to start the reaction. Meanwhile, 10 μL of the buffer were added to the blank wells. Then, it was let rest at 37 °C for 15 min.

The synthetic fluorescence resonance energy transfer peptide utilized in this assay is the usual substrate for renin. It is linked to a fluorophore at one end and a nonfluorescent chromophore at the other. After the peptide is cleaved by renin, the product is highly fluorescent and can be easily analysed by recording the fluorescence intensity (FI) on a fluorescence plate reader (Powerscan HT; BioTek Instruments, Inc., Winooski, VT, U.S.), with an excitation wavelength of 360 nm and an emission wavelength of 528 nm. The analyses were performed in triplicate. The renin inhibitory activity was calculated as follows:
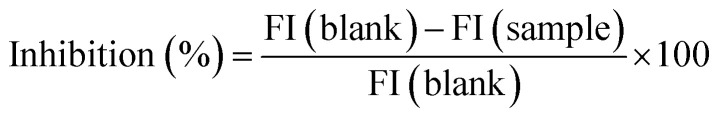
where: FI (blank) was the absorbance of blank, FI (sample) was the absorbance in presence of sample.

### 2.4 Assay for ACE inhibition

The determination of ACE inhibition was performed according to Li *et al.*^29^ The aqueous extract of the samples was appropriately diluted and a 96 well microtiter plate was used as a reaction container. Then, 15 μL of the sample solution (in the control reaction solution, this was replaced with distilled water) was mixed with 30 μL of 4.66 mmol L^−1^ of HHL (previously dissolved in 0.6 mol L^−1^ NaCl–0.4 mol L^−1^ phosphate buffer, pH 8.5). Subsequently, 30 μL of 12.5 mU mL^−1^ ACE enzyme solution were added (for the blank of the sample reaction solution and the control reaction solution, distilled water was used instead), and the reaction was taken to a microplate mixer at 37 °C for 1 h. To stop the enzyme reaction, 120 μL of 1.2 mol L^−1^ NaOH solution were added, followed by 30 μL of 2% OPA solution (dissolved in methanol). The latter was mixed and left resting at room temperature for 20 min. The derivatization reaction was terminated with 30 μL of 6 mol L^−1^ HCl solution. Finally, fluorescence absorption intensity was measured with an excitation wavelength of 340 nm, emission wavelength of 455 nm, and a slit width of 5 nm. The ACE inhibition rate of the samples was calculated as follows:
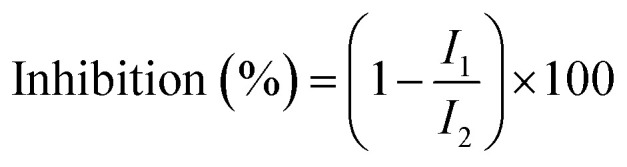
where *I*_1_ indicated the fluorescence absorption intensity of the sample reaction solution in the presence of the ACE inhibitor; and *I*_2_ specified that the control reaction solution had no ACE inhibition.

### 2.5 Determination of total antioxidant capacity

#### 2.5.1 Ferric reducing antioxidant power (FRAP) assay

The FRAP assay was carried out according to the procedure described by Benzie *et al.*^30^ Briefly, TPTZ solution was prepared as follows: 25 mL of 0.3 mol L^−1^ acetate buffer and 2.5 mL of 10 mmol L^−1^ TPTZ working solution were mixed, and then 2.5 mL of a 20 mmol L^−1^ FeCl_3_ solution was added. Successively, 1.8 μL of the TPTZ solution was taken and added to 10 μL of sample extract and mixed with 1 mL of distilled water. The reaction occurred at 37 °C for 10 min. Finally, the absorbance was determined at 593 nm. The standard curve was constructed using Trolox solution (0.03125–1 mg mL^−1^) and the results were expressed as Trolox per g dry of flowers (mg TE per g DW).

#### 2.5.2 DPPH free radical scavenging assay

The antioxidant activities of the samples were analysed by investigating their potential to scavenge the DPPH free radical. For this purpose, the method by Cai *et al.*^31^ was employed with some modifications, as will be described hereon. Samples at a concentration of 5 mg mL^−1^ were evenly mixed with 200 μL of 0.08 mg mL^−1^ DPPH ethanol solution and then incubated for 30 min in the darkness. The absorbance was measured at 517 nm. In the blank control, the same volume of distilled water was used instead of the sample. The final result was expressed as antioxidant capacity, this was Trolox per g dry of flower (mg TE per g DW). The scavenging rate of DPPH was calculated according to the following formula:
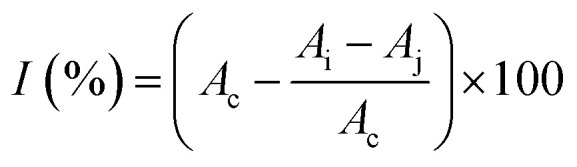
where *I* was DPPH scavenging rate; *A*_c_ was the blank absorbance; *A*_i_ was the sample absorbance; *A*_j_ was the absorbance without DPPH.

#### 2.5.3 Determination of ABTS^+^ scavenging capacity

Antioxidant activity in the samples was analysed by investigating their potential to scavenge the ABTS^+^ radicals. We used the method described by Ozgen *et al.*^32^ Briefly, ABTS reagent was prepared by mixing 5 mL of 7 mmol L^−1^ ABTS stock solution with 88 μL of 140 mmol L^−1^ potassium persulfate and then letting rest in the dark for 12 h. Then, the mixture was diluted with ethanol before measuring absorbance at 732 nm. Finally, 25 μL of the sample extract were added to 2 mL of ABTS solution, and let rest for 6 min in the dark to subsequently determine absorbance. Trolox solution (final concentration 0.03125–1 mg mL^−1^) was used as a reference standard. The results were expressed as Trolox per g dry of flower (mg TE per g DW). The scavenging rate of ABTS was calculated based on the following formula:
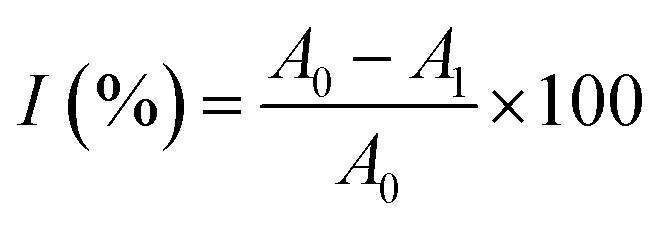
where *I* was ABTS scavenging rate; *A*_0_ was the blank absorbance; *A*_1_ was the absorbance of the test sample.

### 2.6 Contents of total phenolics

Total phenolic content (TPC) was determined by the Folin–Ciocalteu method according to McDonald *et al.*^33^ with minor modifications. Briefly, 100 μL of the sample solution was added to 100 μL of 10% Folin–Ciocalteu solution and incubated at 37 °C. After 10 min, 100 μL of 10% Na_2_CO_3_ was added and the mixture was left to rest at 37 °C for 60 min in the dark. The absorbance was measured at 750 nm. The standard curve was set using various concentrations of gallic acid in distilled water. Total phenolic content (TPC) was expressed as mg gallic acid equivalent (GAE) per g dried weight (DW).

### 2.7 Content of flavonoids

The total content of flavonoids (TF) was analysed using the method described by Pourmorad *et al.*^34^ Briefly, 100 μL of extract were mixed with 625 μL of distilled water and 375 μL of 5% NaNO_2_ solution. After 6 min, 75 μL of a 10% AlCl_3_·6H_2_O solution were added. The mixture was left to rest for another 5 min before the addition of 250 μL of 1 M NaOH. The absorbance was measured at 510 nm using a spectrophotometer. Results were expressed as mg of catechin equivalents per g of dry weight (mg CE per g DW).

### 2.8 Statistical analysis

Data were expressed as means ± standard errors after triplicate evaluation. The statistical software employed was the IBM SPSS Statistics (version 19.0). Duncan's multiple range test was used to evaluate differences among samples. Differences at *p* < 0.05 were considered statistically significant.

## 3 Results and discussion

### 3.1 Renin and ACE inhibitory activities in tree peony petals of different colours and flowering stages

**Fig. 2:**
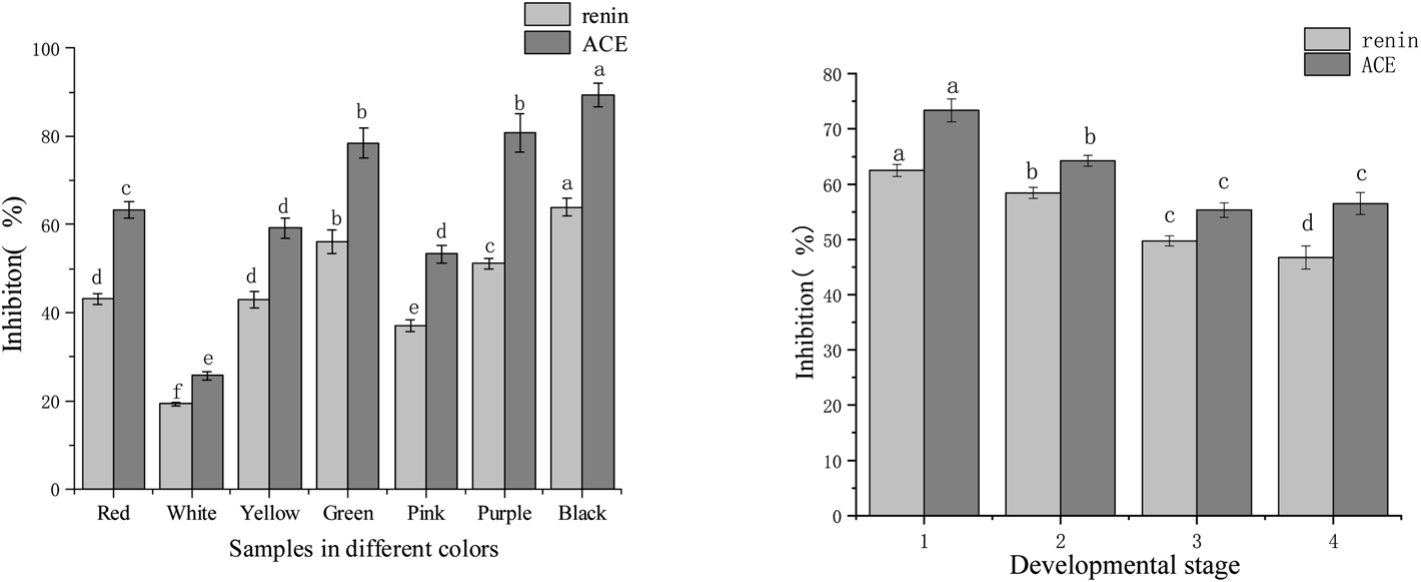
Renin and ACE inhibitory activity in tree peony petals of different colours and flowering stages (different letters indicate significant differences at *p* < 0.05).

The renin inhibitory activity experiment was carried out at a sample concentration of 0.27 mg mL^−1^, while the ACE inhibitory activity test was at a sample concentration of 0.59 mg mL^−1^. Results according to each flower colour were shown in Fig. 2. Seven colour varieties of tree peonies showed significant differences in the inhibitory activities of the two enzymes. The inhibitory order of strength for renin and ACE, respectively, was: black ‘Wujin Yaohui’ (64.00% and 89.49%) > purple ‘Zhao Zi’ (51.20% and 81.74%) > green ‘Dou Lv’ (56.22% and 78.47%) > red ‘Hong Taiyang’ (43.20% and 63.40%) > Yellow ‘Yao Huang’ (43.00% and 59.20%) > pink ‘Zhao Fen’ (37.00% and 53.30%) > white peony ‘Dan Fengbai’ (19.20% and 25.70%). The above indicated that, in tree peony petals, differences in flower colour and corresponding chemical composition significantly influenced the inhibitory ability of renin and ACE.

The different flowering stages of red tree peony ‘Hong Taiyang’ and their relation to renin and ACE were shown in Fig. 2. The inhibitory activities of the two enzymes showed a decreasing trend from flower bud to fully open. In particular, there were significant differences in the first three stages: the inhibition rates of renin and ACE were 62.53% and 73.34% (respectively) in the compact stage of flower bud, 58.45% and 64.30% in the loose stage of flower bud, 49.76% and 55.34% in the half opening stage, and 46.74% and 56.53% when fully open.

In previous studies, we found that the aqueous extract of red tree peony petals had significant inhibitory activity over renin and ACE, with an IC_50_ of 0.08 mg mL^−1^ for renin, and 0.23 mg mL^−1^ for ACE.^27^ Other reported plant extracts that inhibit these enzymes were prepared from *Vernonia amygdalina* (IC_50_ of 0.513 and 0.413 mg mL^−1^ for renin and ACE, respectively),^15^ green tea (IC_50_ of 0.48 mg mL^−1^ for renin^16^), and green soybean (protease hydrolysate; IC_50_ 0.14–1.14 mg mL^−1^ (ref. 35) for ACE). Contrary to these other sources, tree peony petals could be regarded as an important source of natural renin and ACE inhibitory substances. In this study, after comparing the inhibitory activity among flowers of different colours, the results indicated that red, black, purple, green, and yellow tree peony petals had the highest inhibitory activity. Moreover, petals in the bud stage had superior inhibitory activities than those in full bloom.

### 3.2 Total antioxidant capacity in tree peony petals of different colours and flowering stages

**Fig. 3:**
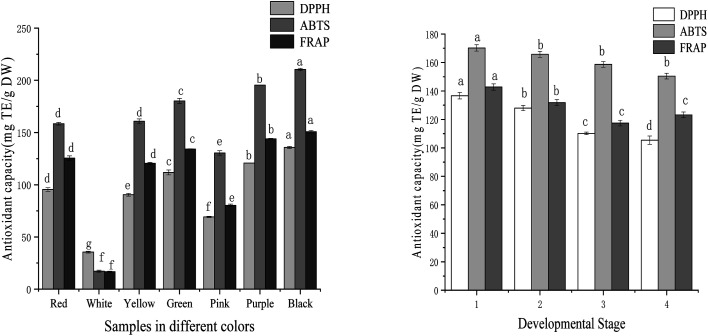
Antioxidant ability in tree peony petals of different colours and flowering stages (different letters indicate significant difference at *p* < 0.05).

DPPH, ABTS, and FRAP analyses were used to evaluate the antioxidant capacity of samples. The antioxidant activities of tree peony petals of different colours and red tree peony ‘Hong Taiyang’ at different flowering stages were listed in Fig. 3. The iron ion reduction ability of tree peony petals of different colours ranged from 16.66 mg TE per g DW to 150.77 mg TE per g DW (9-fold difference). The strongest iron ion reduction capacity was 150.77 mg TE per g DW for Black ‘Wujin Yaohui’ followed by 143.96 mg TE per g DW for purple ‘Zhao Zi’, 134.22 mg TE per g DW for green ‘Dou Lv’, 125.48 mg TE per g DW for red ‘Hong TaiYang’, 80.23 mg TE per g DW for pink ‘Zhao Fen’, and 16.66 mg TE per g DW for white ‘DanFeng Bai’. Red tree peony ‘Hong TaiYang’ showed a decreased trend from bud compaction to full bloom, with 158.43 to 113.24 mg TE per g DW, respectively.

The DPPH free radical scavenging capacity of tree peony petals ranged from 35.47 to 130.78 mg TE per g DW after evaluating different colours (a 4-fold change). The black sample ‘Wujin Yaohui’ had the strongest DPPH free radical scavenging capacity (130.78 mg TE per g DW), followed by purple ‘Zhao Zi’ (120.75 mg TE per g DW), green ‘Dou Lv’ (111.78 mg TE per g DW), red ‘Hong Taiyang’ (95.43 mg TE per g DW), pink ‘Zhao Fen’ (69.22 mg TE per g DW), and white tree peony ‘Danfeng white’ (35.47 mg TE per g DW). The results for the red tree peony ‘Hong Taiyang’ at four different flowering stages showed a decreasing trend from bud compaction to full bloom, ranging from 136.67 mg TE per g DW to 105.43 mg TE per g DW.

The ABTS free radical scavenging capacity ranged from 17.28 to 210.41 mg TE per g DW after evaluating different petal colours (a 10-fold change). The strongest outcome was for black ‘Wujin Yaohui’ (210.41 mg TE per g DW), followed by purple ‘Zhao Zi’ (195.31 mg TE per g DW), green ‘Dou Lv’ (180.24 mg TE per g DW), red ‘Hong Taiyang’ (158.43 mg TE per g DW), pink ‘Zhao Fen’ (130.43 mg TE per g DW), and white ‘Danfeng Bai’ (17.28 mg TE per g DW). Red ‘Hong Taiyang’ petals showed a decreasing trend from bud compaction to full bloom, with 170.24 to 150.43 mg TE per g DW, respectively.

The seven tree peony petals of different colours showed a certain degree of antioxidant capacity, but the difference was significant. The antioxidant capacity order was black > purple > green > yellow > red > pink > white. For red tree peony ‘Hong Taiyang’, this capacity decreased from bud compaction to full bloom. Roses with an important antioxidant capacity have also shown their best antioxidant activity at the bud stage.^36^ The antioxidant properties of tree peony petals are attributed to phenolics and flavonoids.^21^ In the few articles that have also investigated the antioxidant capacity of these flowers at different colours, their results indicated that dark petals had significantly higher values than that of light petals,^21,37^ which agrees with our findings. Given the high antioxidant activity found in tree peony petal extracts, they can be regarded as valuable natural antioxidant sources, and be even applied to food, fragrance, and cosmetic products. It should be noted that the antioxidant capacity of the samples was positively correlated with the inhibition activity over renin and ACE. As displayed in Table 1, the correlation coefficients for inhibition of renin with DPPH, ABTS, and FRAP were 0.98, 0.95, and 0.94, respectively. The correspondent three values for ACE were 0.96, 0.99, and 0.96.

**Table 1:** Correlation analysis between activities and total contents of phenols and flavonoids in different aqueous extracts^a^

Pearson correlation	Renin	ACE	DPPH	ABTS	FRAP	TPC	TF
Renin	1						
ACE	0.99	1					
DPPH	0.98	0.99	1				
ABTS	0.95	0.96	0.96	1			
FRAP	0.94	0.96	0.97	0.98	1		
TPC	0.95	0.96	0.98	0.98	0.933	1	
TF	0.50	0.43	0.75	0.56	0.571	0.95	1

a The difference is significant at the test level of *P* < 0.01.

### 3.3 TPC and TF in tree peony petals of different colours and flowering stages

**Fig. 4:**
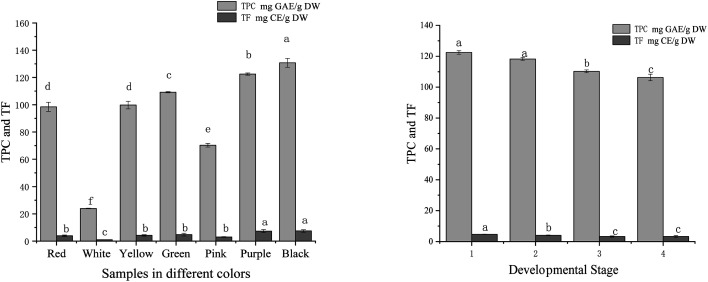
Total phenolic and total flavonoids with different colours and flowering stages of tree peony petals (different letters indicate significant difference at *p* < 0.05).

The TPC and TF in tree peony petals of different colours and red tree peony ‘Hong Taiyang’ petals at different flowering stages were shown in Fig. 4. The content of phenolic compounds (mg g^−1^) in aqueous extracts was expressed in gallic acid equivalents (GAE). The values among different colours ranged from 23.94 to 130.78 mg GAE per g DW (a 4-fold change). The highest TPC was observed in the black petal ‘Wujin Yaohui’ (130.78 mg GAE per g DW), followed by purple ‘Zhao Zi’ (122.45 mg GAE per g DW), green ‘Dou Lv’ (109.22 mg GAE per g DW), yellow ‘Yao Huang’ (99.76 mg GAE per g DW), red ‘Hong Taiyang’ (98.43 mg GAE per g DW), pink ‘Zhao Fen’ (mg 70.32 GAE per g DW), white ‘Danfeng Bai’ (23.94 mg GAE per g DW). The TPC in red ‘Hong Taiyang’ petals at different flowering stages decreased from bud compaction to full bloom (122.45 to 106.23 mg GAE per g DW). The concentration of flavonoids was expressed in catechin equivalents in mg g^−1^ of plant extract. Total flavonoids among different petal colours ranged from 1.02 to 8.89 mg CE per g DW. The values in red tree peony ‘Hong Taiyang’ decreased from bud compaction stage to full bloom stage, from 4.68 to 3.23 CE per g mg DW.

The content of phenols in tree peony flowers was relatively high compared to other plants.^38^ The phenolic compounds in peony flowers were antioxidant, anti-inflammatory, antibacterial, and prolong the life of nematodes.^21,37,39^ Various studies have determined that flowers rich in phenolic compounds can inhibit some chronic diseases.^40,41^ It is worth noting that renin and ACE inhibitory activities have been detected in polyphenols extracted from soybeans (saponins),^14^ black and oolong tea,^16^ tomato,^17^ and flavonoids from rosebuds.^18^ These studies denote that polyphenols can be regarded as potential renin and ACE inhibitors. In this study, we found significant differences among tree peony petals of different colours regarding renin/ACE inhibition capacities and antioxidant activities. All these activities showed significant correlations with polyphenol content (Table 1). The correlation coefficients between total phenol content and renin or ACE inhibition were 0.95 and 0.96, respectively, while the correlation values between total phenol content and DPPH, ABTS free radical scavenging capacity, and iron ion reduction ability were 0.98, 0.98, and 0.99, respectively. These findings support polyphenols as relevant renin and ACE inhibitors.

## 4 Conclusions

This study found that aqueous extracts of red, white, yellow, green, pink, purple, and black tree peony petals had a certain degree of inhibitory effect against renin and ACE. However, the inhibitory activity was significantly different among colours; the order of strength was: black > purple > green > red > yellow > pink > white. In red ‘Hong Taiyang’ petals, the inhibitory activities against renin and ACE decreased from bud compaction to the full opening stage. In addition, a certain degree of antioxidant capacity was detected in the evaluated aqueous extracts, and the samples with higher renin and ACE inhibitory activities also exhibited greater ABTS, DPPH free radical scavenging ability, and iron ion reduction ability. Notably, all the functional activities mentioned above were positively correlated with total phenol content (*p* < 0.01). Our findings evidenced that the phenolic presented in tree peony petals was the main active component responsible for the difference in renin and ACE inhibitory activities. In conclusion, this work provided a broader range of options for the development of renin and ACE inhibitors and a richer theoretical basis to further improve the functional value system of tree peony. For this purpose, it seems necessary to realize its high-value utilization and promote the development of an industry. In the future, more active inhibitors of renin and ACE could be further explored, isolated, purified and identified from aqueous extracts of tree peony petals.

## Conflicts of interest

The authors declare that they have no conflicts of interest.

## Supplementary Material
